# A device for simultaneous neural recording and drug infusion in rodents

**DOI:** 10.1016/j.ohx.2025.e00721

**Published:** 2025-11-12

**Authors:** Giovanni Barbera, Nicholas J. Beacher, Da-Ting Lin

**Affiliations:** a Intramural Research Program, National Institute On Drug Abuse, National Institutes of Health, Baltimore, MD 21224, USA; b Department of Psychology, Wittenberg University, Springfield, OH 455044, USA; c The Solomon H. Snyder Department of Neuroscience, Johns Hopkins University School of Medicine, Baltimore, MD 212054, USA

**Keywords:** Neural recording swivel, Motorized commutator, Drug infusion, Drug self-administration, Miniscope commutator

## Abstract

Here we present and validate an open-source motorized swivel which allows simultaneous neural and behavioral recording from rodents during drug infusion experiments. Cable and drug line tangling represents a limiting factor for neural recordings in freely moving rodents. This limits the scope of experiments that can be carried out using currently available commutators.

The proposed device offers a cost-effective solution to the problem of cable twisting and line tangling during experiments in freely moving rodents, enabling in vivo recordings for new experimental paradigms such as drug self-administration. Additionally, it requires minimal human supervision, simplifying the experimental design pipeline and facilitating scalability.

To demonstrate the usefulness and functionality of the device, we performed in vivo miniscope imaging in rat during a drug self-administration task, showing individual neurons’ response to drug seeking and consumption.

## Highlights

• Motorized commutator for behavior and neural recording during drug infusion.
• Custom 3D printed design and off-the-shelf components make it accessible.
• In vivo miniscope imaging during drug self-administration.

## Graphical abstract

**Figure:**
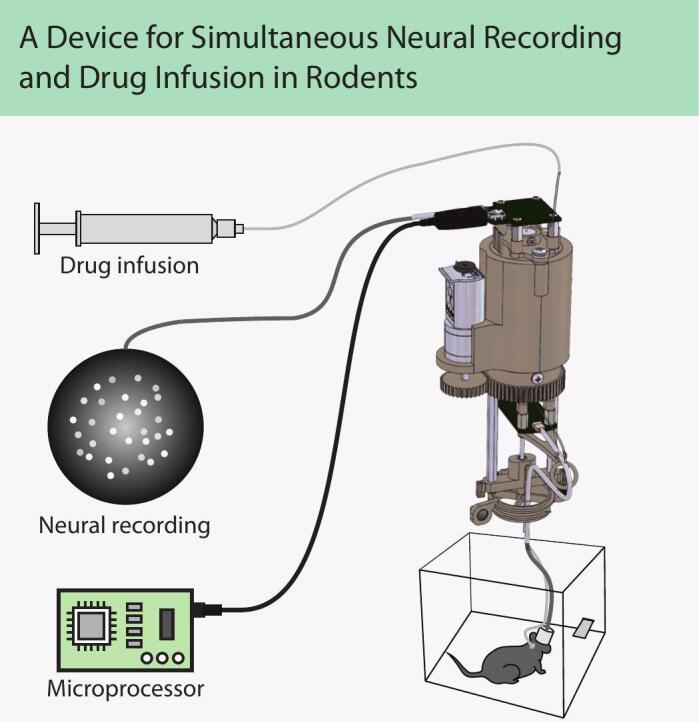

**Specifications table:** 

Hardware name	Motorized commutator for drug infusion and neural recording
Subject area	Neuroscience
Hardware type	Lab interface for neural/behavioral recording and drug infusion
Closest commercial analog	Open-Ephys Torque-Free Commutator – SPI
Open-source license	GNU GPL
Cost of hardware	<1000 USD
Source file repository	https://doi.org/10.5281/zenodo.17435776

## 1 Hardware in context

For recording neural activity during complex behaviors in freely moving rodents, the use of a commutator is often required to manage the tether and avoid equipment failure and experiment interruptions. In the case of small rodents (e.g., mice and rats) passive commutators are challenging to use because the torque required is too large and it would introduce tangling of the tether. To address this issue, several solutions have been proposed in the recent years, both commercially available and open-source, at varying price points and levels of versatility and compatibility, with motor driven active commutators being the most effective solution [[1], [2], [3], [4], [5]]. Compared to existing commercially available commutators, the proposed device offers not only the novel possibility of simultaneous neural/behavioral recording and drug infusion, but also increased flexibility for integration in custom setups, and limited costs, at the expense of a more time consuming building phase, given the intrinsic complexity of the device. For most active commutators, the design hinges around similar principles, where the rotation or twisting of the tether is measured (typically through a Hall effect sensor [1], but other methods, such as video detection, have been proposed [6,7]) and used to control the actuator driving the rotor. These solutions enable in vivo recordings of neural activity (e.g. through electrophysiology, fiber photometry, calcium imaging, or a combination of these methods [4]) for a wide range of complex behaviors. However, to the best of our knowledge, there is currently no active commutator which supports the integration of a drug infusion line together with miniscope imaging. This would allow to monitor neural activity in brain regions of interest during complex behaviors such as drug self-administration [8], which could provide crucial insights in understanding the effect of different drugs in more natural settings, and the neural circuits associated with both drug seeking and response. To address this issue, we propose a 12-channel active commutator which integrates a swivel for a drug infusion line, compatible with 22 ga and 25 ga tubing.

## 2 Hardware description

The commutator design is an improvement on [2], with the integration of a through bore slipring, housing the drug infusion swivel. Small rotations and twisting of the cable and tubing are measured through a Hall effect sensor mounted on the rotor side printed circuit board (PCB), and a control signal is generated by a microcontroller (such as Arduino) and sent to the brushed DC motor controlling the rotor, through the motor driver mounted on the stator PCB. All motor control signals can be accessed through a μHDMI connector on the stator PCB, and the 12 channels are accessible from both the stator and rotor PCBs through 12-pin Omnetics polarized nano connectors (Omnetics Connectors Corporation, Minneapolis MN). The interface was designed to be used with a miniscope [9], but it can be easily adapted to any recording system requiring 10 or less channels (2 of which can be low-voltage differential signaling − LVDS) and 2 power lines. This can be done either by modifying the PCB connectors, or by adding a custom connector for interfacing with the specific recording device and data acquisition hardware. Additionally, all discrete components can be sourced off-the-shelf from major distributors, and mechanical parts can be 3D-printed, keeping the overall building costs contained, and making it accessible to most neuroscience labs.

**Fig. 1:**
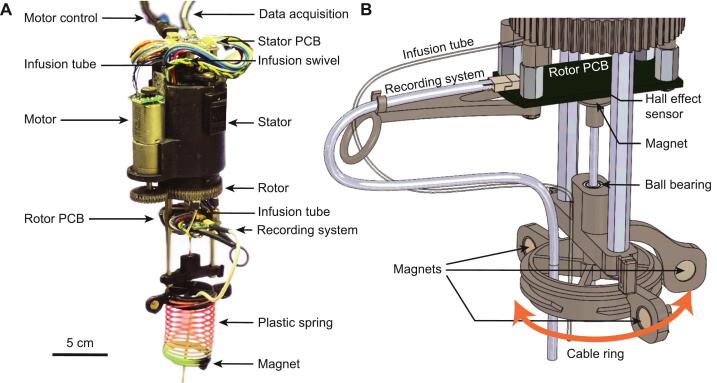
Commutator parts. **A**. Image of the commutator, highlighting the main mechanical components. **B**. Detail of the rotor and cable ring system.

The stator houses the through bore slipring, the drug infusion swivel, the motor, and the stator PCB, connected to the data acquisition system and commutator microcontroller (Fig. 1A). On the rotor side, both the data cable and infusion line pass through a ring which can be easily rotated by the motion of the cable/drug line from the rodent (Fig. 1B). The ring is connected through a shaft to a diametrically magnetized neodymium magnet used to detect its angular displacement with a Hall effect sensor mounted on the rotor PCB. Any detected change in the angle of the cable ring is compensated for by adjusting the rotor position to the new equilibrium.

## 3 Design files summary

**Fig. 2:**
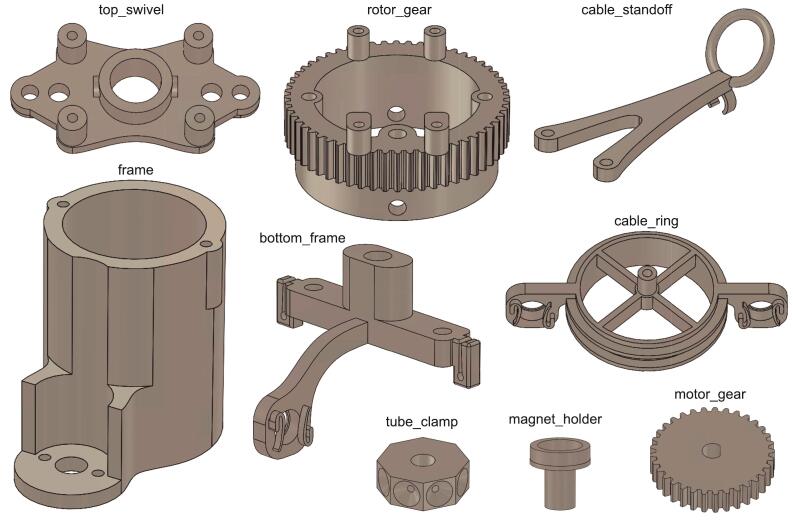
3D printed parts.

All mechanical parts can be 3D printed using consumer grade 3D printers, or through online services (standard ABS, 80 % infill, 100 μm tolerance). The list of parts is shown in Table 1 and Fig. 2, and their function is summarized below.

**Table 1:** design files.

**Design filename**	**File type**	**Open source license**	**Location of the file**
frame.SLDPRT	CAD file	GNU GPL	https://github.com/giovannibarbera/commutator/CAD_files
top_swivel.SLDPRT	CAD file	GNU GPL	https://github.com/giovannibarbera/commutator/CAD_files
rotor_gear.SLDPRT	CAD file	GNU GPL	https://github.com/giovannibarbera/commutator/CAD_files
motor_gear.SLDPRT	CAD file	GNU GPL	https://github.com/giovannibarbera/commutator/CAD_files
tube_clamp.SLDPRT	CAD file	GNU GPL	https://github.com/giovannibarbera/commutator/CAD_files
bottom_frame.SLDPRT	CAD file	GNU GPL	https://github.com/giovannibarbera/commutator/CAD_files
magnet_holder.SLDPRT	CAD file	GNU GPL	https://github.com/giovannibarbera/commutator/CAD_files
cable_ring.SLDPRT	CAD file	GNU GPL	https://github.com/giovannibarbera/commutator/CAD_files
cable_standoff.SLDPRT	CAD file	GNU GPL	https://github.com/giovannibarbera/commutator/CAD_files
stator_PCB.zip	Gerber files	GNU GPL	https://github.com/giovannibarbera/commutator/PCB
rotor_PCB.zip	Gerber files	GNU GPL	https://github.com/giovannibarbera/commutator/PCB

*Frame* – the main body of the commutator, housing the slipring, swivel, motor, and stator PCB.

*Top_swivel* – anchors the swivel to the frame and supports the stator PCB.

*Rotor_gear* – spur gear on the rotor side.

*Motor_gear* – spur gear on the motor side.

*Tube_clamp* – allows to clamp the tubing to the rotor, locking their rotation to avoid tangling.

*Bottom_frame* – houses the ball bearings and the static magnet used to keep the cable ring in neutral position.

*Magnet_holder* – connects the radial magnet used by the Hall effect sensor to the cable ring shaft.

*Cable_ring* – assembly with the two axial magnets moved by the cable.

*Cable_standoff* – used to create a slack and compensate for pulling of the cable.

The stator and rotor PCBs are the same used in [2], and they can be ordered from any PCB manufacturing service using the files provided through the in Table 1. Given the limited number of discrete components, the assembly can be done by hand using soldering iron, using a reflow oven, or it can be outsourced using a PCB assembly service.

## 4 Bill of materials summary

The components required to build the commutator include 3D printed parts, mechanical components, and PCB/discrete electronic components: a complete list is included in Table 2.

**Table 2:** Bill of materials.

**Designator**	**Component**	**Number**	**Cost per unit − USD**	**Total cost − USD**	**Source of materials**	**Material type**
**3D printed parts**						
frame	frame	1	62.50	62.50	https://www.hubs.com	SLA Resin
top_swivel	top_swivel	1	7.03	7.03	https://www.hubs.com	SLA Resin
rotor_gear	rotor_gear	1	14.84	14.84	https://www.hubs.com	SLA Resin
motor_gear	motor_gear	1	3.36	3.36	https://www.hubs.com	SLA Resin
tube_clamp	tube_clamp	1	0.23	0.23	https://www.hubs.com	SLA Resin
bottom_frame	bottom_frame	1	6.25	6.25	https://www.hubs.com	SLA Resin
magnet_holder	magnet_holder	1	0.78	0.78	https://www.hubs.com	SLA Resin
cable_ring	cable_ring	1	4.53	4.53	https://www.hubs.com	SLA Resin
cable_standoff	cable_standoff	1	2.71	2.71	https://www.hubs.com	SLA Resin
**Mechanical components**						
slipring	GT1235-S18	1	275.00	275.00	https://www.moflon.com	Plastic, metal
swivel	375/22	1	246.00	246.00	https://www.instechlabs.com	Stainless steel
metal_gearmotor	3703	1	43.95	43.95	https://www.pololu.com	Other
magnetic_encoder	3499	1	9.95	9.95	https://www.pololu.com	Other
motor_cable	455–3644-ND	1	1.52	1.52	https://www.digikey.com	Metal, Plastic
slipring_cable	455–3723-ND	4	2.91	11.64	https://www.digikey.com	Metal, Plastic
radial_magnet	469–1070-ND	1	0.60	0.60	https://www.digikey.com	Neodymium
signal_cable	455–3723-ND4	4	2.91	11.64	https://www.digikey.com	Metal, plastic
axial_magnet	5862 K406	3	1.06	3.18	https://www.mcmaster.com	Neodymium
rod	3180 T31	1/2 ft	9.92	9.92	https://www.mcmaster.com	Stainless steel
sleeve	50415 K54	0.5 *m*	8.51	8.51	https://www.mcmaster.com	Stainless steel
ball_bearings	7804 K119	2	7.03	14.06	https://www.mcmaster.com	Stainless steel
standoff_short	91075A515	8	3.79	30.32	https://www.mcmaster.com	Stainless steel
standoff_long	93655A227	2	5.68	11.36	https://www.mcmaster.com	Stainless steel
washers	92141A008	1 pack	1.53	1.53	https://www.mcmaster.com	Stainless steel
screws_6-32_7/16	91772A149	1 pack	6.82	6.82	https://www.mcmaster.com	Stainless steel
screws M3x10	92000A120	1 pack	6.22	6.22	https://www.mcmaster.com	Stainless steel
screws_2-56_1/4	91772A077	1 pack	6.47	6.47	https://www.mcmaster.com	Stainless steel
screws_M2.5x8	92000A105	1 pack	4.74	4.74	https://www.mcmaster.com	Stainless steel
setscrews_2-56_1/8	92311A074	1 pack	13.35	13.35	https://www.mcmaster.com	Stainless steel
plastic_spring	B0DKBRPYC3	1 pack	7.99	7.99	https://www.amazon.com	Plastic
**PCB**						
stator_PCB	stator_PCB	1	20.20	20.20	https://www.pcbway.com	Other
U16	A79623-001	1	29.00	29.00	https://www.omnetics.com	Other
C5	311–3365-1-ND	1	0.18	0.18	https://www.digikey.com	Other
C1 C4	399–8269-1-ND	2	0.43	0.86	https://www.digikey.com	Other
U1	455–1806-1-ND	1	0.83	0.83	https://www.digikey.com	Other
JP2 JP1	455–1809-1-ND	2	0.98	1.96	https://www.digikey.com	Other
C2 C3	490–3261-1-ND	2	0.10	0.20	https://www.digikey.com	Other
R1	541–1888-1-ND	1	0.10	0.10	https://www.digikey.com	Other
U4 U5	BSS138CT-ND	2	0.31	0.62	https://www.digikey.com	Other
Q1	DMP2045U-7DICT-ND	1	0.43	0.43	https://www.digikey.com	Other
R5 R15 R6 R16	P10KGCT-ND	4	0.10	0.40	https://www.digikey.com	Other
IC1	TB6612FNGC8ELCT-ND	1	1.95	1.95	https://www.digikey.com	Other
U14	WM1281CT-ND	1	2.66	2.66	https://www.digikey.com	Other
rotor_PCB	rotor_PCB	1	19.80	19.80	https://www.pcbway.com	Other
U3	A79623-001	1	29.00	29.00	https://www.omnetics.com	Other
LED1	160–1423-1-ND	1	0.30	0.30	https://www.digikey.com	Other
JP3 JP4	455–1809-1-ND	2	0.98	1.96	https://www.digikey.com	Other
C6	490–3261-1-ND	1	0.10	0.10	https://www.digikey.com	Other
U2	AS5030-ATSTCT-ND	1	5.38	5.38	https://www.digikey.com	Other
U6	BSS138CT-ND	1	0.31	0.31	https://www.digikey.com	Other
R4	P1.00KHCT-ND	1	0.10	0.10	https://www.digikey.com	Other
R2 R3	P10KGCT-ND	2	0.10	0.20	https://www.digikey.com	Other
Total cost				943.54		

## 5 Build instructions

**Fig. 3:**
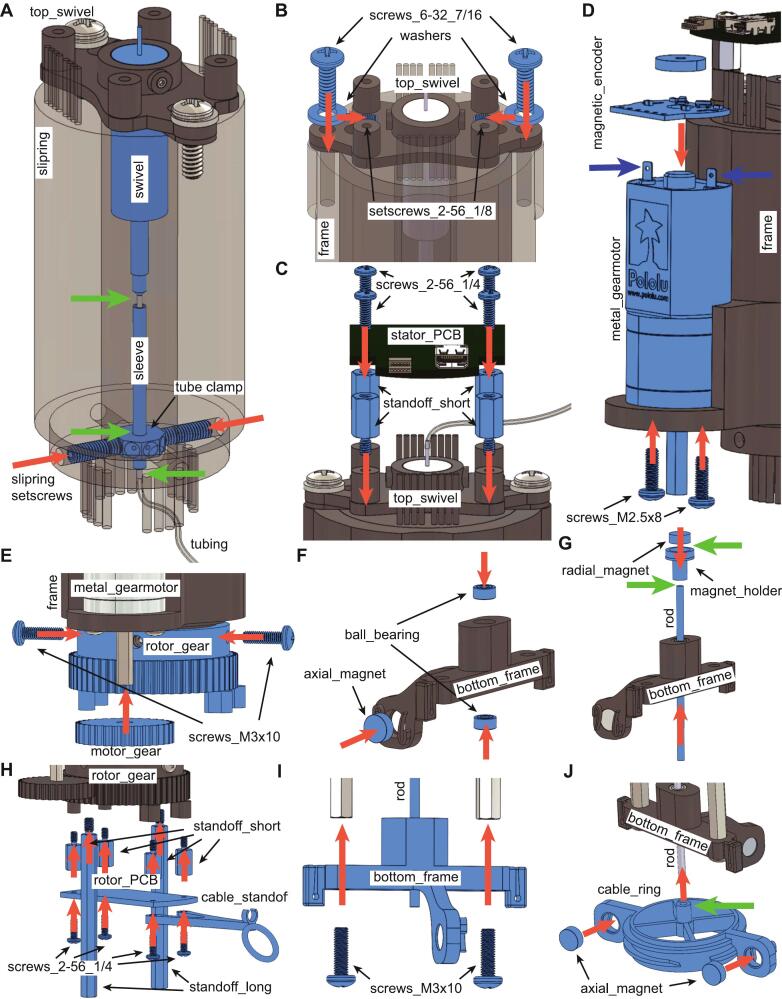
Building steps for assembling the commutator. Parts mounted at each step are highlighted in blue; red arrows indicate component installation direction, green arrows indicate location where to apply epoxy, and blue arrows indicate where to apply solder. (For interpretation of the references to colour in this figure legend, the reader is referred to the web version of this article.)

**Fig. 4:**
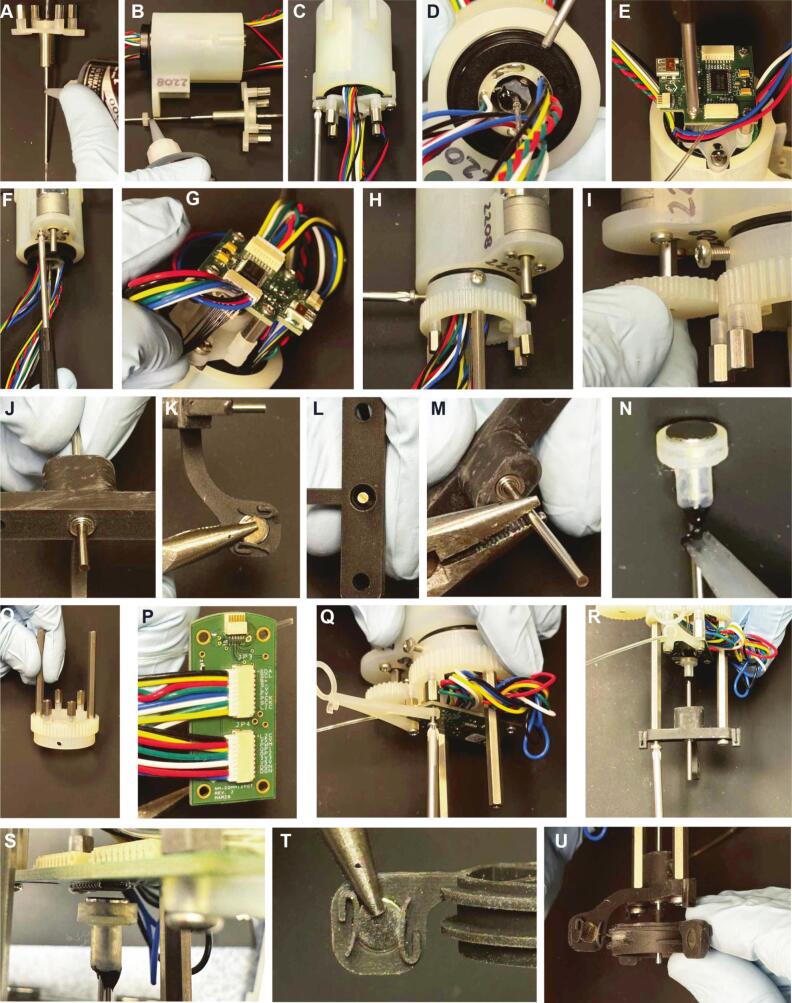
Pictures of the mechanical assembly steps.

The building steps to assemble the commutator are outlined below and depicted in Fig. 3 and Fig. 4.

### 5.1 Mechanical assembly steps

Step 1 (Fig. 3A): prepare the *swivel* before sliding it in the commutator *frame*, by inserting the 22/25 ga tubing (∼6 in long) on the rotor side; then slide the metal *sleeve* over the tubing and apply epoxy where it connects to the swivel (Fig. 4A). Insert the *tube_clamp* over the sleeve, aligning it to the 4 screw holes on the slipring’s rotor (Fig. 4B) and secure it to the metal sleeve with epoxy.

Step 2 (Fig. 3B): secure the *swivel* to the *top_swivel* piece using the 2 *setscrews_2-56_1/8*, then attach *top_swivel* to *frame* with *screws_6-32_7/16* and *washers* (Fig. 4C). Use two *slipring_setscrews* on opposite sides (as shown in Fig. 3A and Figure Fig. 4D) to secure *tube_clamp* to *slipring*.

Step 3 (Fig. 3C): mount 4 *standoff_short* to *top_swivel* and install *top_PCB* with *screws_2-56_1/4* (Fig. 4E).

Step 4 (Fig. 3D): solder *magnetic_encoder* to motor leads and install *metal_gearmotor* to *frame* with *screws_M2.5x*8 (Fig. 4F). Connect slipring and motor to stator PCB as shown in Fig. 4G.

Step 5 (Fig. 3E): slide *rotor_gear* over rotating part of the slipring with 4 *screws_M3x10* (two of them will be on the *slipring_setscrews*, do not overtighten, see Fig. 4H). Insert *motor_gear* on motor shaft (Fig. 4I).

Step 6 (Fig. 3F): mount the 2 *ball_bearings* (Fig. 4J) and *axial_magnet* (Fig. 4K) on *bottom_frame*. Insert rod through *ball_bearings*, adjusting their alignment if needed to ensure a smooth rotation (if rod does not rotate smoothly, most likely at least one of the *ball_bearings* is tilted and needs to be aligned, Fig. 4L-M).

Step 7 (Fig. 3G): Use epoxy to attach *radial_magnet* to *magnet_holder* and to end of *rod* (Fig. 4N).

Step 8 (Fig. 3H): mount 4 standoff_short and 2 *standoff_long* on *rotor_gear* (Fig. 4O); connect *slipring* with *rotor_PCB* as shown in Fig. 4P, then use 4 *screws_2-56_1/4* over each *standoff_short* to install *rotor_PCB* and *cable_standoff* (Fig. 4Q).

Step 9 (Fig. 3I): secure *bottom_frame* to the 2 *standoff_long* using *screws_M3x10* (Fig. 4R). Adjust the position of the *rod* such that the gap between *radial_magnet* and the Hall effect sensor *U2* is 1 mm (Fig. 4S).

Step 10 (Fig. 3J): mount 2 *axial_magnets* to *cable_ring*, aligning them with magnetic north pointing in opposite directions, and matching (Fig. 4T). Slide *cable_ring* on *rod* until magnets are aligned (Fig. 4U), and secure with epoxy.

### 5.2 PCB assembly steps

For the PCB assembly and connections the procedure is similar as in [2]. The small number of surface mount components makes the PCB assembly process accessible to most labs, requiring only a minimal number of tools.

All PCB artwork files, bill of materials, stencils and component positioning files can be found at https://github.com/giovannibarbera/commutator/tree/master/PCB, including files with PCB requirements for fabrication.

Step 1: Using a needle or small tip, apply solder paste to all pads on one side.

**Fig. 5:**
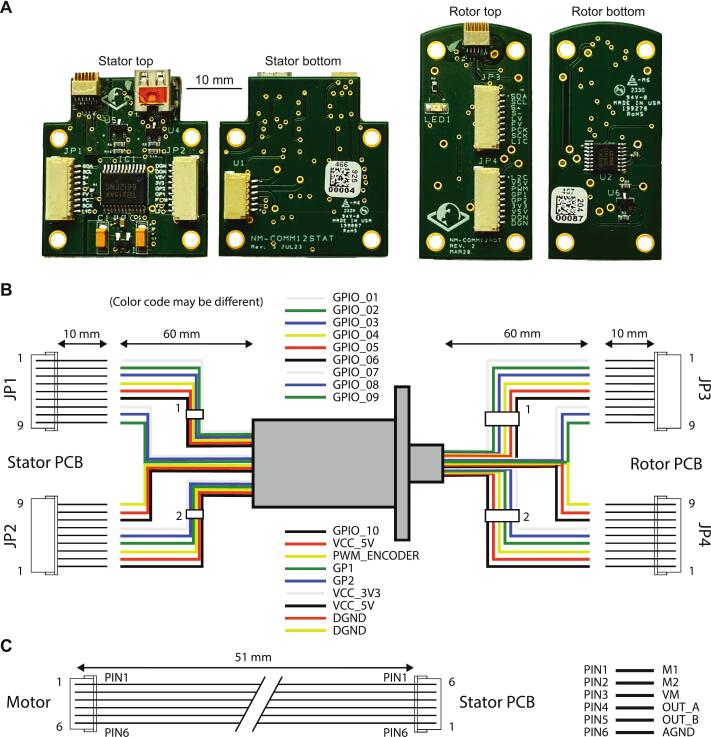
PCB assembly and connections. **A.** Pictures of the assembled stator (left) and rotor (right) PCBs. **B.** Schematic of the slipring connections to the 4 JST 9-pin connectors. **C.** Motor cable connections to the stator PCB.

Step 2: Following the component positioning reference (Fig. 5A, https://github.com/giovannibarbera/commutator/tree/master/PCB), place all components in their respective position, ensuring good contact with solder paste.

Step 3: Keeping component in place with a tweezer, melt the solder paste to the pads with the.

soldering iron, one component at a time. When done, repeat steps 1–3 for the other layer.

Step 4: Clean solder paste residue with a cleaning agent such as isopropyl alcohol and a small brush.

Step 5: Apply a layer of non-conductive epoxy between connectors and PCB, taking care not to spill over on the contacts, to strengthen their resistance to mechanical stress.

After assembling the rotor and stator PCBs, the slipring should be connected to the 4 JST 9-pin connectors as indicated in the schematics in Fig. 5B, and the motor should be connected to the stator PCB following the wiring shown in Fig. 5C.

## 6 Operation instructions

The commutator can be used in many experimental setups, using both commercially available or custom mice or rat chambers with minimal required modifications. The main consideration when designing a set up with the motorized commutator is the top clearance and vertical space requirements, especially in the case of stacked chambers or when using overhead cameras.

Vertical space requirements are typical of 7″ for the commutator plus 12″-18″ for the cable. It is advisable to use a plastic spring attached to the cable ring and anchor the cable/tubing assembly to the bottom of the spring with a magnet, as shown in the assembly in Fig. 1A: this reduces the slack on the cable, allowing the rodent to reach all corners of the chambers and limits the risk of tangling or the rodent being able to grasp the cable.

The control algorithm of the commutator is robust and straightforward, which makes it suitable for being integrated into many types of recording systems with a simple custom interface such as in [9].

**Fig. 6:**
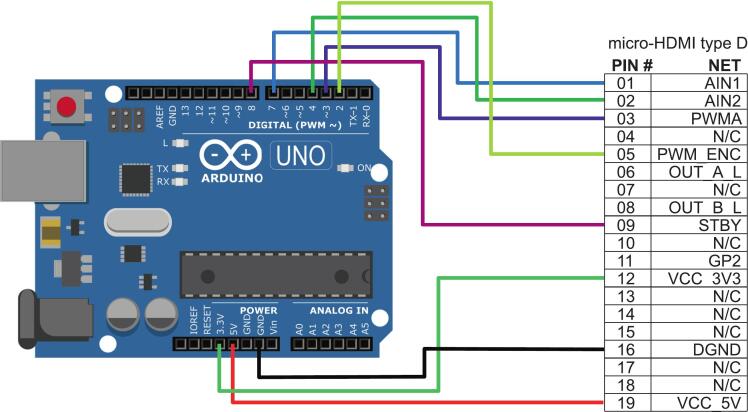
Schematic of the connections to Arduino Uno.

Alternatively, the commutator can be operated with a microcontroller, such as Arduino Uno, independently from any recording device (similar to [2]), requiring minimal attention during experiments and no need for parameter tuning. The schematics with the connections to Arduino Uno are shown in Fig. 6, and can be adapted to similar microcontrollers. Since the motor driver (Toshiba TB6612FNG) is already mounted on the stator PCB, minimal wiring is required for the microcontroller, which simply needs to supply the motor control signal. A sample control program can be found at https://github.com/giovannibarbera/commutator.

One important aspect of the control algorithm is the initial calibration at power on, which determines the control position of the magnet. To ensure the cable ring is in neutral position during the power on calibration, a binder clip can be used to keep the cable ring aligned with the bottom frame for the first few seconds.

### 6.1 Troubleshooting

Here we discuss some common problems which could be encountered during assembly or operation of the motorized commutator.

*Problem*: Motor gear slipping.

*Solution*: Insert a metal or plastic wedge between the commutator body and the motor to keep the motor shaft closer to the rotor.

*Problem*: Motor not functioning.

*Solution*: It could be generally either due to a connection issue (check that both the 6-pin motor connector and all 4 9-pin connectors are crimped and connected properly), or a configuration issue (make sure the motor is working properly by testing a sample motor control program). If the test control program works but the commutator still does not rotate, check that the magnet is close enough to the Hall effect sensor (a green LED light indicates that the magnet is detected).

*Problem*: Sata from recording device is noisy, only while the motor is running.

*Solution*: Add 0.1μF ceramic capacitors across motor terminals.

*Problem*: Data from recording device is noisy.

*Solution*: In many cases data transmission issues arise from the 9-pin connectors. Test all 4 connectors and, if needed, rewire the ones with connection issues. Epoxy can also be used on the connectors after crimping, to avoid any possible damage caused by forces applied to the wires.

*Problem*: The cable ring assembly does not rotate smoothly.

*Solution*: The cause is likely the two ball bearings not being aligned. First slide the top ball bearing on the rod and mount it on the bottom frame; move the rod to make sure it is perfectly centered. Slide in the bottom ball bearing and push it in its slot. Use pliers to ensure it sits perfectly flat.

*Problem*: Tubing seems clogged.

*Solution*: If it cannot be flushed using a syringe, the tubing is likely twisted near the swivel connection. This could happen if the tubing detaches from the swivel or the tube clamp. Replace the tubing and use a stronger epoxy to secure it to swivel and tube clamp.

*Problem*: Motor rotates constantly in one direction.

*Solution*: This could be caused by the Hall sensor losing contact with the radial magnet, usually when pulling on the cable causes the rod to slide through the ball bearings. Add a small drop of epoxy between the rod and the bottom ball bearing, being careful not to spill into the ring.

## 7 Validation

We tested the motorized commutator by running a drug self-administration task (Movie S1, Movie S2) on 3 rats (2 female and 1 male) while simultaneously recording single cell neural activity in the Nucleus Accumbens (NAc) using a miniature fluorescence microscope [10] in custom operant chamber [11].

### 7.1 Results

**Fig. 7:**
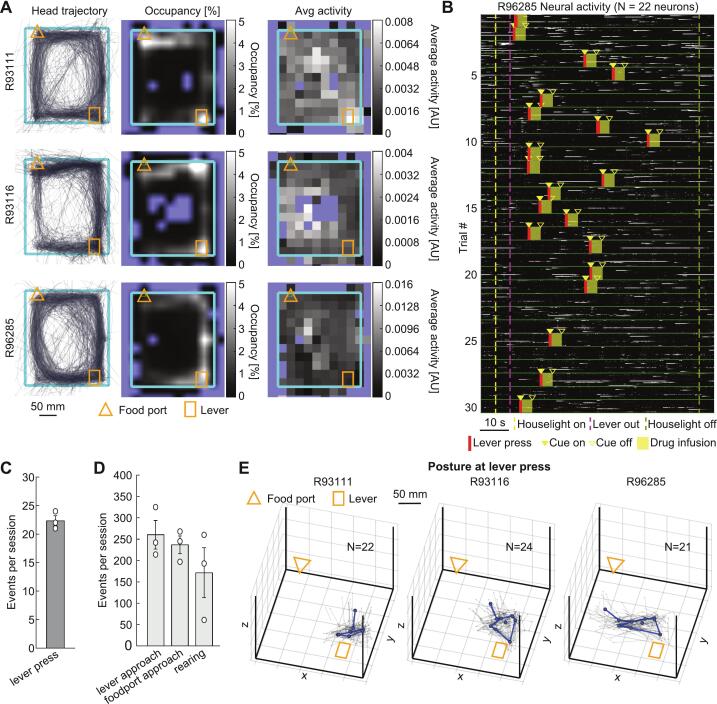
Single cell imaging during drug self-administration. **A**. x-y representation of the head trajectory (left column), occupancy map for the head position (middle column) and average activity from all neurons (right column). Each row represents a different rat. **B.** Rasterplot showing the activity of 22 neurons detected on a representative rat for all the 30 trials, separated by green lines. Most relevant behavior events are overlaid. **C.** Number of lever presses per session. **D.** Number of other representative behavior events per session. **E.** 3D view of the postures displayed by each rat at the onset of each lever press bout (grey lines represent single bouts, thick blue line represents average). (For interpretation of the references to colour in this figure legend, the reader is referred to the web version of this article.)

For characterizing the motorized commutator, we recorded neural activity from individual neurons during a 30-trial drug self-administration session (Fig. 7, Movie S3).

We first observed behavioral data indicating sustained locomotor activity, lever pressing, and food port/lever-directed movement. These behaviors are characteristic of active drug-seeking in rats and were consistently recorded during the experiment. These findings are illustrated in Fig. 7A-B, which shows the behavioral tracking trajectories and the corresponding calcium traces rasterplots. Rats were actively lever pressing throughout the session (22.33 +/- 1.53 standard deviation (SD) trials with lever press out of 30 trials, Fig. 7C), and performing other typical behaviors such as rearing and moving towards the food port/lever location (Fig. 7D) − even though no food was delivered during this experiment.

Although the overall neural activity did not significantly increase at any particular location (Fig. 7A, right), we further investigated whether individual neurons exhibited tuning to specific behavioral events. To this end, we estimated the 3D position of 15 body parts for each frame by triangulating their 2D coordinates obtained from multiple camera views. This data was then used to identify specific behaviors (e.g. rearing, food port/lever approach/leave, freezing, left/right turns, etc.) in an unsupervised fashion, and to identify body postures during these behaviors. For instance, we observed that each rat had a consistent body posture during lever press across bouts (Fig. 7E), however, this posture varied significantly across individual rats, suggesting personalized behavioral patterns that may correspond to distinct neural activity profiles.

**Fig. 8:**
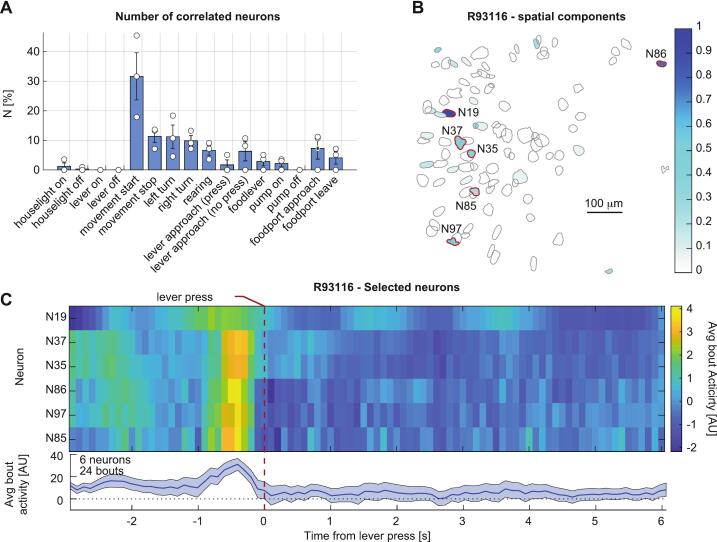
Neural tuning to behavior. **A.** Size of the ensemble tuned to each of the selected behaviors, expressed as percentage of each rat total number of detected neurons. **B.** Cell map for representative rat: color intensity represents the average *peri*-event activity (+/- 1 s) around lever press. **C.** Rasterplot of average activity across lever press bouts for the 6 neurons highlighted in red in **B** (top), and their average (bottom). (For interpretation of the references to colour in this figure legend, the reader is referred to the web version of this article.)

NAc neurons have been shown to play a crucial role in motivated behaviors, reward and aversion, which are critical aspects of drug seeking and substance use disorders (SUDs) [[12], [13], [14]]. However, technical limitations have hindered the ability to track the activity of individual neurons during drug self-administration. With the proposed system we were able to identify individual neurons in the NAc showing significantly higher activity during specific behaviors (Fig. 8A), such as lever press (Fig. 8B-C), suggesting the involvement of the rat’s NAc in drug self-administration task, and the importance of studying single cell activity to understand the neural encoding of relevant behaviors such as drug seeking.

Following a similar line of research, and taking advantage of transgenic rodent lines and recent advances in miniscope development [15] (such as dual color imaging [16,17] or simultaneous imaging and optogenetic stimulation [18]), it will be possible isolate cell type-specific neuronal circuits involved in drug seeking, expanding our understanding of how neuronal ensembles encode drug reward, and ultimately providing possible targets for developing selective treatments for substance use disorders (SUDs).

Altogether, these preliminary results suggest that the use of the proposed commutator does not hinder the ability of the rat to perform complex tasks such as food and drug self-administration, locomotion, grooming and exploration, enabling the study of individual neurons’ long-term activity in complex behaviors. In future applications, this system could be implemented more broadly to study neural activity patterns in any brain region in response to specific drugs or pharmacological interventions, simply adapting the connection interface to match the recording device being used.

### 7.2 Stereotaxic surgery

Virus: cre-dependent AAV-FLEX-jGCaMP8m (Addgene: 162381) is injected into the Nucleus Accumbens of 2 female and 1 male rats (1750 nl total, 50 nl/minute, 15 min rest, Titer = 4.89x10^12) using coordinates for males (ML = 1.6, AP = 1.6, DV = 6.7 with a 0.1 mm ‘hole’ for virus to sit in) and females (ML = 1.6, AP = 1.6, DV = 6.6 with a 0.1 mm ‘hole’ for virus to sit in) using a blunt tip nanofil syringe.

**Fig. 9:**
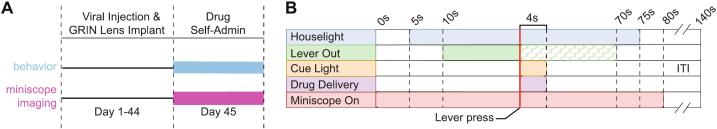
**A.** Experiment timeline. **B.** Drug self-administration trial timeline.

Lens: a 9 mm long, 1 mm diameter GRINTECH lens is implanted slowly (speed = 0.22 mm/minute) and then fixed to the skull using dental cement. The recording session is carried out 45 days after viral injection (Fig. 9A).

### 7.3 Catheterization

Following confirmation of fluorescence, we then catheterize the rat. The rat is anesthetized using isoflurane gas (5 % induction, 1–2 % maintenance) and surgery begins when the rat fails to show a reflex in response to a toe pinch. We repeat the ‘toe pinch’ throughout the procedure to confirm proper anesthetic levels throughout the procedure. We shave the surgical areas (entire back area + area near right jugular vein) and scrub 3 times alternating with betadine scrub and 70 % alcohol. We then place the rat on its stomach and make a 2 in. incision (perpendicular to the rat’s lengthwise body) on the center of the back near the bottom of the ribcage. We then make a smaller 1 in. incision in the center of the back near the shoulder blades (also going perpendicular to the rat’s body). We then flip the rat on its back and make a 1 in. incision (going parallel to the rat’s body lengthwise) and blunt dissect to expose the jugular vein. We then use silk sutures to prop the jugular vein up against tissue. The wound is covered with sterile gauze, and we then turn the rat on its stomach and pass the catheter subcutaneously through the body and out of the area near the exposed jugular vein. The IV connector is secured into the smaller exposed incision protruding from the back (pushing it from the larger incision and popping it out of the smaller one). We rotate the rat onto its stomach and close these wounds with suture and clean the area with hydrogen peroxide. Post surgery we flush the catheter with gentamicin (4.25 mg/ml, 0.1 ml) and close the IV connector with a sterilized plastic cap to protect the connector. We require this surgery to be performed after confirming the success of the lens implant surgery to minimize the risks associated with leaving an implanted catheter during the lens implant recovery period (lasting ∼ 30 days).

### 7.4 Methamphetamine self-administration

Rats then self-administered methamphetamine (0.04 mg/ infusion over 4 s) via a lever press under a fixed-ratio-1 (60 s timeout) reinforcement schedule in the presence of a 70 s house light cue stimulus for 30 trials total (Fig. 9B). Trials begin with the onset of a white house light followed 5 s later by the extension of a lever which is a discriminative stimulus (DS) for methamphetamine availability. Following a single lever press, the lever retracts, a secondary light cue is active for 4 s and is accompanied by the automatic infusion of methamphetamine through the catheter. Trials end 65 s after house light onset and is followed by a 60 s intertrial interval (ITI).

### 7.5 Calcium data analysis

The raw videos recorded with the miniscope were first motion corrected using a custom Matlab algorithm based on Fast Fourier Transform (FFT); a 3x3 median filter was then applied to the images for noise reduction. Neural traces and spatial footprints were extracted from the registered videos for each of the 80 s trials using CaImAn [19], and neural footprints were registered across trials [20].

### 7.6 Behavior video analysis

The video streams from the 2 overhead cameras, located on opposite corners of the box, were synchronized with the miniscope data and behavior timestamps using transistor-transistor logic TTL signals. The median pixel value across frames for each 80 s trial was calculated and used to manually identify landmark points in the box and estimate the position and orientation of the cameras given their intrinsic parameters. Behavior video frames were processed with DeepLabCut [21] to identify the position of 15 body parts, and, using the camera calibration data, the 3D position of each body part was estimated using triangulation.

## Ethics statements

All the experimental procedures and animal care included in this paper comply with the ARRIVE guidelines and were approved by the Institutional Animal Care and Use Committee (ACUC), the Intramural Research Program (IRP), National Institute on Drug Abuse (NIDA), National Institutes of Health (NIH), and performed in accordance with the guidelines of NIH NIDA IRP ACUC.

## CRediT authorship contribution statement

**Giovanni Barbera:** Writing – review & editing, Writing – original draft, Visualization, Validation, Software, Project administration, Methodology, Formal analysis, Data curation, Conceptualization. **Nicholas J. Beacher:** Writing – original draft, Methodology, Investigation. **Da-Ting Lin:** Writing – review & editing, Writing – original draft, Supervision, Investigation, Funding acquisition, Conceptualization.

## Funding

This research was supported by the Intramural Research Program of the National Institutes of Health (NIH). The contributions of the NIH author(s) are considered Works of the United States Government. The findings and conclusions presented in this paper are those of the author(s) and do not necessarily reflect the views of the NIH or the U.S. Department of Health and Human Services.

## Declaration of competing interest

The authors declare that they have no known competing financial interests or personal relationships that could have appeared to influence the work reported in this paper.
